# Regionalizing muscle activity causes changes to the magnitude and direction of the force from whole muscles—a modeling study

**DOI:** 10.3389/fphys.2014.00298

**Published:** 2014-08-13

**Authors:** Hadi Rahemi, Nilima Nigam, James M. Wakeling

**Affiliations:** ^1^Neuromuscular Mechanics Laboratory, Department of Biomedical Physiology and Kinesiology, Simon Fraser UniversityBurnaby, BC, Canada; ^2^Department of Mathematics, Simon Fraser UniversityBurnaby, BC, Canada

**Keywords:** differential activity patterns, aponeurosis stiffness, finite element method, muscle model, lateral gastrocnemius

## Abstract

Skeletal muscle can contain neuromuscular compartments that are spatially distinct regions that can receive relatively independent levels of activation. This study tested how the magnitude and direction of the force developed by a whole muscle would change when the muscle activity was regionalized within the muscle. A 3D finite element model of a muscle with its bounding aponeurosis was developed for the lateral gastrocnemius, and isometric contractions were simulated for a series of conditions with either a uniform activation pattern, or regionally distinct activation patterns: in all cases the mean activation from all fibers within the muscle reached 10%. The models showed emergent features of the fiber geometry that matched physiological characteristics: with fibers shortening, rotating to greater pennation, adopting curved trajectories in 3D and changes in the thickness and width of the muscle belly. Simulations were repeated for muscle with compliant, normal and stiff aponeurosis and the aponeurosis stiffness affected the changes to the fiber geometry and the resultant muscle force. Changing the regionalization of the activity resulted to changes in the magnitude, direction and center of the force vector from the whole muscle. Regionalizing the muscle activity resulted in greater muscle force than the simulation with uniform activity across the muscle belly. The study shows how the force from a muscle depends on the complex interactions between the muscle fibers and connective tissues and the region of muscle that is active.

## 1. Introduction

Skeletal muscles can contain subunits called neuromuscular compartments that are spatially distinct regions that contain specific motor units and motor drive from the nervous system (English et al., 1993). In muscles with broad attachments, a relationship between anatomical compartments and function may appear logical, and this has shown to be the case for both the biceps femoris in the cat (English and Weeks, 1987; Chanaud et al., 1990) and the masseter muscle in the pig (Herring et al., 1989, 1991). However, functional regionalization in muscles with long tendons has also been reported (Carrasco et al., 1999), leading to the suggestion that activation of motor units in different compartments may result in differences to both the direction and the magnitude of force applied at the tendon (English et al., 1993). It is likely that asymmetry in the fascicle architecture combines with the location of the neuromuscular compartments to result in varied force vectors from a contracting muscle.

Unipennate muscle is asymmetrical in its architecture, and muscle fibers in different locations have different moment arms and may exert different torques about a joint. The way in which forces are transmitted from the contractile fibers to the tendon can involve myofascial pathways (Huijing, 2003), that in turn may modify the resultant force vector from the individual fibers. It has been shown that activity can differ between the neuromuscular compartments and spatial regions in the gastrocnemii in the cat during walking (English, 1984), and in man during both cycling and postural tasks (Wakeling, 2009; Hodson-Tole et al., 2013). Changes in activity between the neuromuscular compartments in the cat lateral gastrocnemius led to changes in the direction of the force vector along the tendon, altering the moments of yaw, pitch or roll about the ankle (Carrasco et al., 1999). However little has been reported about the mechanisms that link the varied forces developed by individual fibers to the net mechanical output of the whole muscle.

The different stiffnesses of connective tissues such as aponeurosis and tendon add to the complexity of muscle-tendon unit (MTU) behavior. *In-vitro* measurements of mechanical properties of tendon and aponeurosis (Wren et al., 2001; Azizi et al., 2009; Lake et al., 2010), and ultrasound-based *in vivo* measurements of these properties (Maganaris and Paul, 2000; Magnusson et al., 2003) suggest that tendon and aponeurosis may have different tensile elastic moduli which can be alterd with age (Onambele et al., 2006), training (Kubo et al., 2002) and injury (Arampatzis et al., 2007). The elastic properties of the aponeurosis can affect the extent to which muscle fibers rotate as they contract (Randhawa et al., 2013), and can thus affect the magnitude and direction of the forces developed by the whole muscle. However, it is beyond current experimental techniques to measure the effect of aponeurosis stiffness on the force outputs from muscle.

Some of the limitations in experimental studies can be addressed using *in silica* models of muscles. These models need to contain a realistic architecture and physiologically relevant connective tissue properties. Further, they should be able to support different activation levels in different regions. Implementing fundamental physiological concepts and material properties within sophisticated mathematical frameworks has moved muscle simulations from one-dimensional models (Zajac, 1989; Delp et al., 1990) to more architecturally and functionally detailed two—(Van Leeuwen and Spoor, 1992; Otten and Hulliger, 1994) and three-dimensional models (Oomens et al., 2003; Blemker et al., 2005; Bol and Reese, 2008). Despite the level of architectural details that current models include, such models rarely include other heterogeneities within the muscle such as material distribution (e.g., fiber-type or connective tissue properties) or differential patterns of activation. In this study we investigate how uneven patterns of activation across a unipennate muscle affect the magnitude and direction of the force developed by the whole muscle, using a conceptual *in silica* muscle mode. The *in silica* model has a simple geometry but is assymetric in architecture, and regionalized in activity. This model was also used to investigate how the aponeurosis properties affect the force development in the fibers. and its transmission to the external tendon.

## 2. Materials and methods

A unipennate muscle model was created *in silica* to test the effects of the activation being regionalized on the direction and magnitude of the force developed by the whole muscle. The model had a regularized shape to help constrain model variants and results to the conceptual questions which are the focus of this study, rather than allowing the model to respond to idiosyncrasies of individual geometries. The dimensions, as well as the structural and material properties of the model were styled to be consistent with those of the lateral gastrocnemius in humans. The soft tissues were treated as transversally isotropic hyperelastic materials. The muscle-aponeuroses complex was meshed by a grid of hexahedral elements. Displacements, stresses and forces were calculated using a three-field finite element formulation. The activation levels of the different muscle fibers within the model could be independently varied. The methods for each of these parts are described in more detail below.

### 2.1. Geometry, mesh, boundary, and fiber architecture

**Figure 1:**
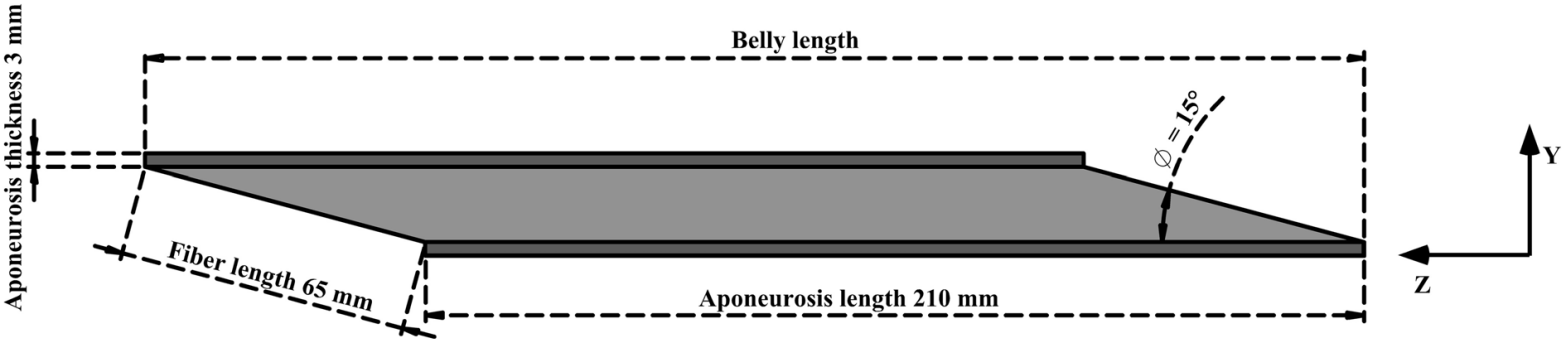
**Simplified geometry of lateral gastrocnemius.** Aponeurosis tissue in dark gray and muscle tissue in light gray. The origin of the muscle coordinate system was set to the bottom right corner of the deep aponeurosis, and the axes were aligned with x, width; y, thickness, z, belly length.

A simplistic geometry (Figure 1) was developed for human lateral gastrocnemius (LG) (Dimensions mostly from Randhawa et al., 2013). For the purpose of this study, the muscle belly was modeled without the attached external tendon. This allowed the belly to remain isometric and constant between conditions. The cross-section of the undeformed muscle belly was a parallelogram. The short side of this parallogram was 65 mm, the same as the length of a muscle fiber. The smaller angle within the parallelogram was 15°, which was also the the angle a fiber made with the aponeurosis at both ends of attachment. This could be considered as the initial pennation in the muscle. However, the angle between the fiber and the line of action of the muscle was smaller. This is because the line of action was calculated to be the diagonal of the parallelogram. The initial width of the muscle belly was set at 55 mm.

Aponeuroses covered the two long sides of the muscle tissue. Each aponeurosis was 210 mm in length and a constant thickness (depth) of 3 mm, with a width matching that of the muscle belly.

Integration points in the *in silica* model were assigned material properties appropriate to aponeurosis or muscle fibers, relative to their position in the belly. The geometry was fixed at one opposing end of each aponeurosis and the other boundary surfaces were traction free.

### 2.2. Material properties of the *in silica* muscle model

For purposes of completeness and setting notation, we first describe the mathematical background for our model. The soft tissues (muscle and aponeurosis) in this project were mathematically described by a fiber-reinforced composite material (Spencer, 1984). Specifically, they are described as nearly incompressible (e.g., for muscle Baskin and Paolini, 1967), transversally isotropic hyperelastic materials. If we denote the position vector of a point in the deformed tissue by **x**, it can be expressed in terms of the position vector of the same point in the undeformed configuration, **X**, and the displacement vector **u** in the natural way: **x** = **X** + **u**. The response of a nearly-incompressible material to loading can be decomposed into a volume-changing (*volumetric*) and a volume-preserving (*isochoric*) part. Specifically, if $F\;:=\;\left[\frac{\partial x_{i}}{\partial X_{j}}\right]$ denotes the usual deformation gradient (a second-order tensor) and *J* : = *det*(*F*) denotes the dilation, then we can define the isochoric part of the deformation gradient as *F*:

(1) $$\bar{F}=\frac{1}{J^{1/3}}F.$$

The deformation gradient is used to calculate the left Cauchy-Green tensor (the Finger tensor) *B* and the Lagrangian finite strain tensor *E* as:

(2) $$B:=FF^{T}=\left[\frac{\partial x_{i}}{\partial X_{k}}\frac{\partial x_{j}}{\partial X_{k}}\right],\;E:=\frac{1}{2}(F^{T}F-I).$$

Using the decomposition in (1), we can obtain the isochoric part of the left Cauchy-Green tensor, which we denote as *B*:

(3) $$\bar{B}:=\bar{F}{\bar{F}}^{T}=\frac{1}{J^{2/3}}FF^{T}=\frac{1}{J^{2/3}}B.$$

As is standard for hyperelastic materials undergoing finite deformations, the mechanical response is described in terms of the strain energy density function *W*. As with the deformation gradients, this strain energy function can be decomposed into its volumetric and isochoric parts:

(4) $$W(B)=W_{vol}(J)+W_{iso}(\bar{B}).$$

The volumetric component *W*_*vol*_ of the strain energy describes the nearly incompressible behavior of the tissues,

(5) $$W_{vol}(J):=\frac{\kappa }{4}\left(J^{2}-1-2\log(J)\right).$$

The volumetric strain energies of muscle and the aponeurosis are distinguished by different choices of the (constant) bulk modulus, κ. In our experiments we used κ = 10^6^ Pa for muscle, and κ = 10^8^ Pa for the aponeurosis.

The isochoric contributions to the strain energy *W*_*iso*_ are described in terms of one (Yeoh, 1993) or more (Blemker et al., 2005) invariants (*I*_1_…*I*_5_) of the left Cauchy-Green tensor *B*, the initial orientation of fiber in the tissue (**a**_0_) and the along-fiber stretch λ:

(6) $$\begin{array}{l} I_{1}:=tr(B),I_{2}:=\frac{1}{2}\left[{\left(tr(B)\right)}^{2}-tr(B^{2})\right],I_{3}:=det(B)=J^{2},\\ I_{4}:=a_{0}\cdot B\cdot a_{0}=\lambda^{2},I_{5}:=a_{0}\cdot B^{2}\cdot a_{0}. \end{array}$$

Using these invariants we can describe the *along fiber isochoric strain energy* (*W_tissue_*) and the *base material isochoric energy* (*W_base_*):

(7) $$W_{iso}=W_{tissue}+W_{base}.$$

The contribution of the base material to the strain energy, *W_base_*, encapsulates the elastic properties of the connective tissue within both muscle and the aponeurosis (e.g., the extracellular connective tissue in muscle). These contributions are mathematically modeled as approximations of an exponential fit for the first invariant *I*_1_ to real data. Both the muscle and the aponeurosis possess their own distinct base material properties. In this paper, the base material isochoric energy is described using a model originally due to Yeoh (1993), which is cubic in *I*_1_:

(8) $$\begin{array}{l} W_{base,muscle}:=\sum_{i=1}^{3}c_{i}{\left(I_{1}-3\right)}^{i},c_{1}=6.75\times 10^{-3}\text{Pa},\\ \;c_{2}=2.78\times 10^{-2}\text{Pa},c_{3}=-1.9745\times 10^{-3}\text{Pa}. \end{array}$$

The base material isochoric energy for the aponeurosis is described using a Humphrey (exponential) model (Humphrey and Yin, 1987),

(9) $$\begin{array}{l} W_{base,apo:=}c_{1}\left(e^{c_{2}(I_{1}-3)}-1\right),c_{1}=4.3510\times 10^{4}\text{Pa},\\ \;c_{2}=5.796\times 10^{2}\text{Pa}. \end{array}$$

The isochoric strain energy contributions, *W*_*tissue*_, arise from the stretching of fibers along their length. If we denote the Cauchy stress in the fiber caused by a stretch of λ as σ_*tissue*_(λ), then the isochoric strain energy for both muscle and aponeurosis becomes

(10) $$\lambda \frac{\partial W_{tissue}(\lambda )}{\partial \lambda }=\sigma_{tissue}(\lambda )$$

Muscle and aponeuroses tissues are distinguished by the specific form of their Cauchy stress σ_*tissue*_(λ) used in (10). For this study, we constructed stress-stretch relationships from experimental data (see below), using the curve-fitting functions in (MATLAB, 2014).

**Figure 2:**
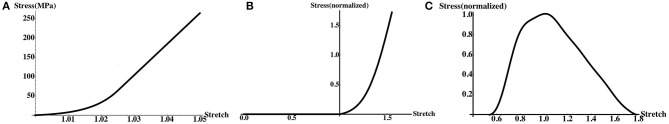
**Along-fiber stress-stretch curves used by the model. (A)** Aponeurosis (Equation 11). **(B)** Passive muscle (Equation 13). **(C)** Active muscle (Equation 16).

For the aponeurosis, σ_*tissue*_ ≡ σ_*Apo*_ was obtained by a piecewise exponential fit of stress-stretch curves from Magnusson et al. (2003) where human gastrocnemius aponeurosis tensile properties were measured. See Figure 2A on page 5.

(11) $$\sigma_{Apo}(\lambda )=\{\begin{array}{l} 10^{6}\times 3.053(\lambda^{124.6}-1)\text{Pa},\;1\leq \lambda \leq 1.025,\\ \begin{array}{l} 10^{6}\times 3.053[17374.99\\ (\lambda -1.025)+20.687]\text{Pa}, \end{array}\;1.025<\lambda . \end{array}$$

Muscle fibers can be passively or actively stretched, and we can therefore decompose σ_*Muscle*_(λ) = σ_*active*_(λ) + σ_*passive*_(λ). We can also express the active and passive stresses normalized by the maximum isometric stress σ_0_ = 200 KPa at the optimal length of the fiber. Experimental data is commonly given in terms of normalized stresses,

(12) $$\sigma_{muscle}(\lambda )=\sigma_{0}\left({\hat{\sigma }}_{Active}(\lambda )+{\hat{\sigma }}_{Passive}(\lambda )\right)$$

The normalized passive stress-stretch relation, $\hat{\sigma }$_*Passive*_(λ), was based on a piecewise exponential fit (Figure 2B) of the experimental data from Zajac (1989).

(13) $${\hat{\sigma }}_{Passive}(\lambda )=\{\begin{array}{l} 0\;\lambda \leq 1.0,\\ 10^{-5}(38.495e^{5.339\lambda }-7945)\;1.0<\lambda . \end{array}$$

To obtain the (normalized) stress-stretch relation $\hat{\sigma }$_*Active*_(λ) for active muscle, we used a Hill-type model (Hill, 1938) of muscle fiber contraction. If we denote the normalized activation level by α(t) which ranges from 0 to 1, and the normalized stress-stretch rate by ${\hat{\sigma }}_{\dot{\lambda }}$, then the (normalized) stress-stretch relation for the active fiber is given by

(14) $${\hat{\sigma }}_{Active}(\lambda )=\alpha (t){\hat{\sigma }}_{\lambda }{\hat{\sigma }}_{\dot{\lambda }}$$

In fact, in order to define different activation levels in different regions of the muscle, we modify (14) to make the dependence on location explicit:

(15) $${\hat{\sigma }}_{Active}(X,\lambda )=\alpha (X,t){\hat{\sigma }}_{\lambda }{\hat{\sigma }}_{\dot{\lambda }}$$

In the simplest instance, we can set α(**X**, *t*) = 0 for inactive regions, and linearly increasing over the simulation time from zero to a selected maximum level of activity α_*max*_ in the remaining. However, this may result in abrupt transitions in activity within the muscle, which may not be physiological. Instead, we used combinations of *arctan* (**X**) functions to vary activity smoothly between regions which were active and those which were not.

In this study, we allowed α(**X**, *t*) to vary linearly from 0 to α_*max*_ of 0.2 during 0.4 s of simulation. In order to describe $\hat{\sigma }$_λ_, we used experimental data from Gordon et al. (1966). In this experiment, the stresses were measured for fully active and isometric muscle fibers with isometric contractions where ${\hat{\sigma }}_{\dot{\lambda }}$ = 1 at steady state. We fit the experimental data with a trigonometric polynomial (Figure 2C) in order to capture the complexity of the data while allowing for smooth derivatives.

(16) $$\begin{array}{l} {\hat{\sigma }}_{\lambda }=0.534+0.229\cos(\omega \lambda )-0.095\cos(2\omega \lambda )\\ \;+\;0.024\cos(3\omega \lambda )-0.021\cos(4\omega \lambda )+0.013\cos(5\omega \lambda )\\ \;-\;0.421\sin(\omega \lambda )+0.079\sin(2\omega \lambda )-0.029\sin(3\omega \lambda )\\ \;+\;0.013\sin(4\omega \lambda )+0.002\sin(5\omega \lambda ), \end{array}$$

with ω = 4.957.

### 2.3. Numerical methods

A three-field finite element formulation was used to solve for the displacement **u**, internal pressure $\tilde{p}\;:=\;\frac{\delta W_{vol}(\tilde{J})}{\tilde{J}}$ and the dilation constraint $\tilde{J}$ = *J*(**u**). We obtained these quantities by using the principle of stationary potential energy, i.e., minima of

(17) $$\begin{array}{l} U(u,\tilde{J},\tilde{p})=\int_{\Omega }W_{vol}+\tilde{p}(J(u)-\tilde{J})dv+\int_{\Omega }W_{iso}\bar{B}(u)dv\\ \;-\int_{\Omega }{\text{f}}_{b}\cdot udv-\int_{\partial \Omega }{\text{f}}_{t}\cdot uda \end{array}$$

Here Ω, ∂Ω, v and *a* are the system's domain, boundary, volume and boundary area respectively and **f***_b_*, **f***_t_* are body and traction forces acting on the domain and boundary of the system respectively. In this study the applied body force **f***_b_* ≡ 0.

We used a discontinuous Galerkin method for **u**, $\tilde{p}$ and $\tilde{J}$. The resultant non-linear system was solved using Newton-Raphson iterations, and the linear solves within each Newton step were performed using a preconditioned conjugate gradient method. The discretization and solution was performed within the deal.II finite element library (Bangerth et al., 2013), and is a modification of a code (http://www.dealii.org/developer/doxygen/deal.II/step_44.html) by Pelteret and McBride to compute the material response of a neo-Hookean material.

### 2.4. Numerical simulations

A set of simulations were designed to investigate the effect of regionalized activation, as well as different aponeurosis stiffnesses, on the magnitude and the direction of force developed by an isometrically activated muscle. The average activation of the whole muscle tissue was set to be 10% but the distribution of activation was changed between the simulations. For initial undeformed (relaxing) state, the muscle fibers were considered to be at optimal length and along-fiber strain in aponeurosis was set to zero.

The different distributions of activation are shown in Table 1. All activation distribution scenarios were repeated for three different elasticity moduli for aponeurosis. The aponeurosis was considered with maximum strains of 2, 5 and 10 % when the muscle was developing maximum isometric force, where the 5% case is given in Equation 11.

**Table 1:** **Level and regionalization of activation in different simulations**.

**Activation pattern**	**Uniform**	**Proximal-distal**	**Midline**	**Medial-lateral**
First view	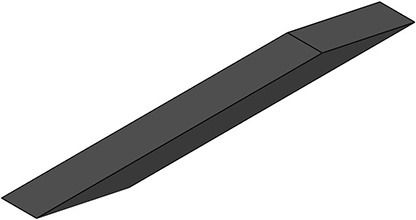	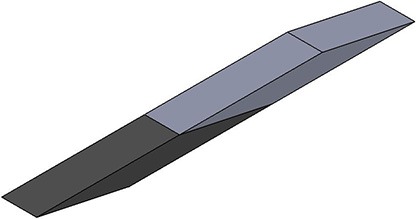	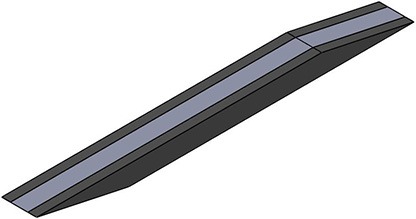	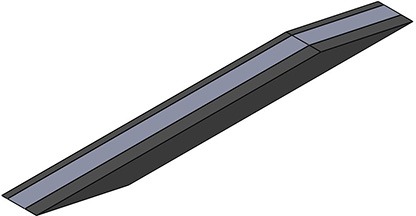
(Second view)	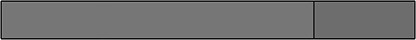	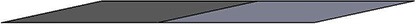	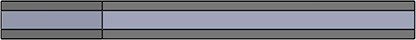	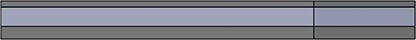
α_*max*_	0.1	0.2	0.2	0.2

*For heterogeneous patterns, the light gray region was activated to the prescribed maximum level (last row), while the dark gray region(s) are inactive. Note that for the medial-lateral activity pattern, the region of activity was not symmetric about the mid-plane (x = 27.5 mm) but instead was offset to one side, to be symmetrical about the plane x = 32.1 mm*.

The model was run on an eight-core machine with multi-threading over the cores (16 threads). The average CPU time for each simulation was approximately 10 min. This included the time needed to initialize the mesh, assemble matrices and iteratively solve the system.

## 3. Results

**Figure 3:**
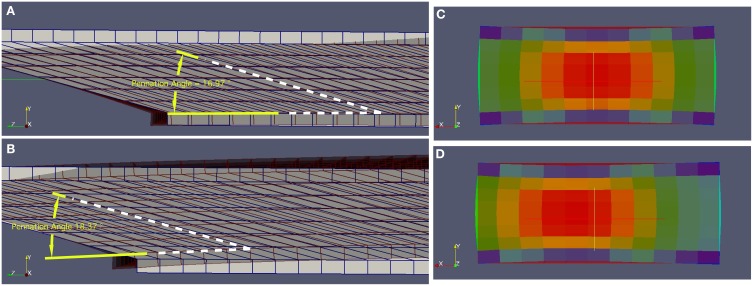
**Deformed (active) and undeformed (relaxed) geometries for (A) the Uniform activation pattern and (B) the proximal-distal activation pattern.** These geometries are shown with pale areas and blue lines for the undeformed states, and darker areas and gray lines for the deformed states. Note that in the deformed states the pennation angle for the proximal-distal activation pattern (18.37°) is larger than for the Uniform activation pattern. Transverse sections through the muscles are shown for the **(C)** Midline activation pattern, and **(D)** Medial-lateral activation pattern. In these panels the undeformed shape is shown by the rectangular and dark red area. The colored elements show the magnitude of the strain in the model tissues in their deformed state, ranging from low strains (blue) in the aponeurosis to greatest strains (red) in the muscle belly. Note how the muscle belly thickness between the aponeuroses is least over the active region of fibers, and the width of the muscles has increased beyond the undeformed state. Also note that in the Medial-lateral activation pattern the maximum strains have moved laterally (to the left) within the muscle.

**Figure 4:**
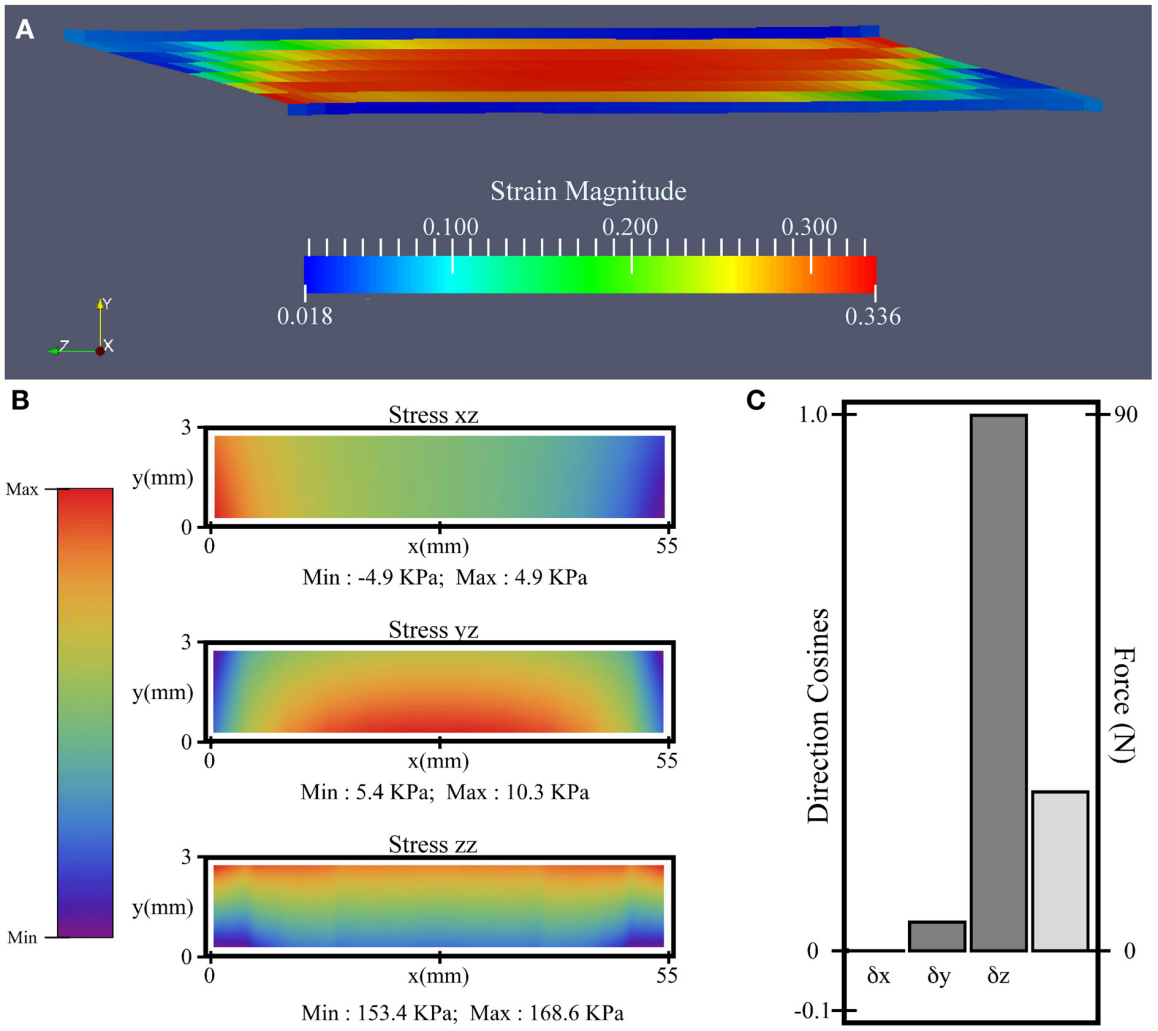
**Simulation results for the uniform activation condition with compliant aponeurosis and 10% activation. (A)** Magnitude of strain. **(B)** “xz” and “yz” shear, and “zz” tensile stress contours on the plane connecting the aponeurosis and tendon (*z* = 0). **(C)** The direction cosines (dark gray) and force magnitude for the resultant force (light gray) acting on the *z* = 0 plane.

During the isometric contractions simulated in this study, the aponeuroses stretched, allowing the muscle fibers to shorten and rotate to greater pennation angles (Figure 3) than the initial value of 15°. A common end-point for the contractions was defined as the time where there was a mean 10% activation across the muscle tissue. The end-point of a contraction can be seen in Figure 4A for the condition with a uniform activation and compliant aponeurosis; this figure shows the total strain for each element in the tissues. However, note that the maximum along-fiber strain in the muscle was 26%. For this condition the aponeurosis stretched up to 1.5%, and the greatest shortening of the muscle fibers occurred in the center of the muscle belly. The muscle belly bulged in its width (x-direction) by approximately 12%, and decreased in the thickness between the aponeuroses. The muscle fibers curved during contraction, with the greatest curvatures occurring close to the aponeusoses, and the fibers following S-shaped paths. The initial condition had the fibers arranged in plane (parallel to the yz plane), and these curved outwards as the muscle belly width increased during contraction.

**Figure 5:**
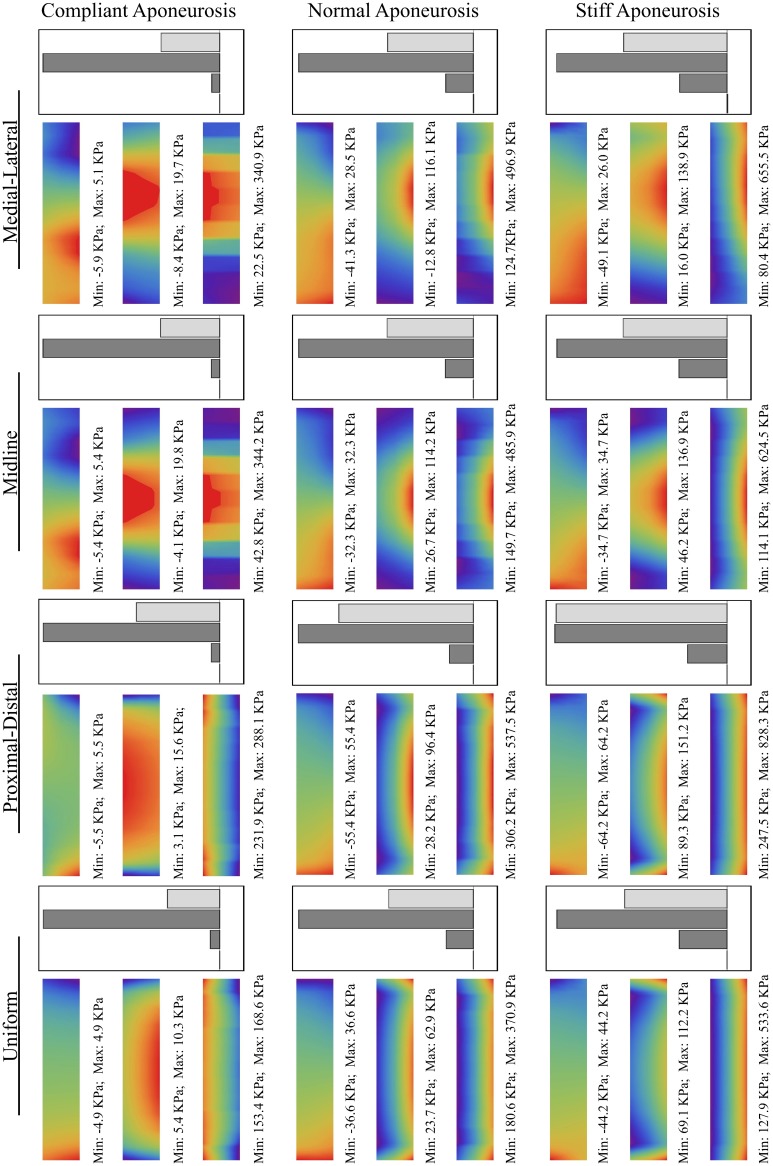
**Stress contours and force magnitudes and directions for the 12 test conditions.** The scales are shown in Figures 4B,C.

The results comparing all 12 simulations can be seen in Figure 5. The force vectors for the whole muscle were calculated from the shear and tensile stresses developed across the transverse plane bounding the deep aponeurosis (*z* = 0, for contour details see Figure 4B). The force vectors were described by the x-, y- and z- direction cosines of the force vector (δx, δy, and δz, respectively), and the resultant force magnitude (for details see Figure 4C).

In general, an increase in aponeurosis stiffness caused an increase in the magnitude of force and a change to its direction (see δy in Figure 5). The stretch in the aponeurosis was reduced for increased aponeurosis stiffness, and this led to a reduction in the shortening of the muscle fibers and a reduction in their rotation to higher pennation angles. Additionally, as the aponeurosis stiffness increased, the changes in muscle belly thickness and width became smaller. For the example of the uniformly activated muscle with a stiff aponeurosis, the width increased by only 7% during contraction.

The conditions with uniform activation had each muscle fiber activated to 10%. For the other 4 conditions with regionalization of the activity, the mean activity level across the muscle was kept at 10%, but this was concentrated in half the fibers each being activated to 20%. The magnitude of the muscle force was similar for the simulations with uniform, midline and medial-lateral distributions of activity, however the proximal-distal activation pattern resulted in greater muscle force.

The active fibers in the conditions with heterogeneous activation patterns (proximal-distal, midline and medial-lateral) contracted to a shorter length and rotated to a greater pennation angle than the uniform pattern. Additionally, in the conditions with proximal-distal activation patterns the thickness of the belly changed non-uniformly along the length of the muscle with a greater reduction in thickness in the active region.

For the compliant conditions, the y-component of the center of force moved from a position midway down the aponeurosis to a level closer to the deep surface for the stiff aponeurosis condition (Table 2). Conditions with the uniform, proximal-distal and midline activation patterns are all symmetrical about the midplane of the muscle (*x* = 27.5 mm). For these conditions there was a negligible x-component to the whole-muscle force, with δx < −10^−6^. The medial-lateral condition has the region of activity displaced to one side (Table 2), with the activity centered about the plane *x* = 32.1 mm. The x-component for the whole-muscle force was increased for this condition (δx ≅ −3 × 10^−3^): this value was still small, however, there was a more substantial increase in the x-component of the center of force (Table 2) acting at the end of the aponeurosis (to *x* = 31.3 mm).

**Table 2:** **x and y components of the center of Force (COF) on the *z* = 0 plane**.

		**COFx (mm)**	**COFy (mm)**
Compliant aponeurosis	Uniform	27.5	1.56
	Proximal-distal	27.5	1.52
	Midline	27.5	1.54
	Medial-Lateral	31.6	1.54
Normal aponeurosis	Uniform	27.5	1.26
	Proximal-distal	27.5	1.30
	Midline	27.5	1.26
	Medial-lateral	30.9	1.26
Stiff aponeurosis	Uniform	27.5	1.06
	Proximal-distal	27.5	1.10
	Midline	27.5	1.07
	Medial-lateral	31.4	1.07

## 4. Discussion

The *in silica* isometric contractions of the lateral gastrocnemius in this study show decreases in muscle fiber length and increases in pennation (Figures 4A,B) in a similar manner to *in vivo* reports (Maganaris et al., 1998). However, the *in silica* muscle belly thickness decreased during contraction in a manner more representative of *in vivo* measurements from the medial gastrocnemius (Maganaris et al., 1998). The simulated muscle belly thickness decreased because a component of the contractile force of the muscle fibers acts to compress the muscle between the aponeuroses, and this was balanced by increases in the width of the muscle belly (Figures 4C,D) to maintain the incompressibility of the muscle tissue. Compressing the muscle belly would cause increases in intramuscular pressure, and this in turn drives increases in the curvature of the muscle fibers (Van Leeuwen and Spoor, 1992). Indeed, in our simulations the initial configurations of the fibers were straight, and this changed to curved S-shapes during contraction. These emergent features of the muscle fibers are consistent with recent reports of S-shaped fascicle trajectories that develop *in vivo* during isometric contractions of the medial gastrocnemius (Namburete et al., 2011). The width of the simulated muscle belly increased during contractions more than the width of the aponeuroses (Figures 4C,D), and so the belly bulged outwards to the sides. A consequence of this bulging was that the planes across which the fibers were initially aligned (parallel to the yz plane) transformed to curved sheets during contraction. Sejerested and co-workers (Sejersted et al., 1984) observed curved fascicle sheets in the vastus lateralis of human cadavers and have suggested the arrangement of fascicle sheets in an onion-like arrangement, and recent 3D reconstructions of fiber curvatures have also demonstrated that the fiber trajectories in both the medial and lateral gastrocnemius curve around concentric sheets during contraction (Rana, 2012). Despite the simplistic initial geometry (Figure 1) of the muscle in these simulations with straight fibers and rectangular aponeuroses, an emergent feature from the simulations was for the fibers to develop curved trajectories in 3D, and this has been suggested to be an important property to maintain mechanical stability within the muscle (Van Leeuwen and Spoor, 1992).

All the *in silica* conditions in this study had an equivalent level of muscle activity with an average of 10% activity across the muscle fibers (see Table 1) in the contracting state. However, both the magnitude and direction of the force (Figure 5) varied with the different regionalizations of the muscle activity. All conditions with regionalized activity resulted in greater muscle forces than for the condition with uniform activity across all fibers, and they also showed localized decreases in thickness, fascicle length and larger pennations around their active regions. These results are consistent with findings by Otten and Hulliger (1994) who compared the level of force in a uniformly fully active muscle with muscles where half the fibers were fully active: they activated the fibers in one end of the muscle and noted that the muscle force was 14% greater than expected from the average activity level. There was a pronounced increase in the muscle force for the non-uniform pattern of activation in the proximal-distal model (Figure 5); this could be due to specific changes in the shape of the muscle belly compared to other activity patterns, where the active region is decreasing in thickness more than the inactive region, and the muscle is bending around the region where the activity levels transitioned. We additionally show that changing the regionalization of the activity results to changes in the direction of the force vector from the whole muscle (Table 2), and this would result in different directions of the muscle torque in the joints that it spans, explaining experimental observations for the lateral gastrocnemius in the cat (Carrasco et al., 1999).

The increases in aponeurosis stiffness in these *in silica* contractions resulted in decreased aponeurosis stretch and muscle fiber strain, and smaller increases in pennation of the fibers. These changes resulted to increases in the magnitude of the force (Figure 5) developed by the whole muscle. These results are consistent with previous finite element models of the biceps femoris in which aponeurosis dimensions were changed with conditions that had stiffer aponeuroses, resulting in reduced fiber strains (Rehorn and Blemker, 2010). In another study, a finite element model of the medial gastrocnemius from the cat demonstrated that increased aponeurosis stiffness resulted in greater muscle forces for isometric contractions (Lemos et al., 2008), again consistent with our results. The combined findings from these three modeling studies show that the interactions between the muscle fibers and the connective tissue are important for shaping the mechanical output from the whole muscle. Our simulations additionally demonstrate how the direction of the muscle force is affected by the stiffness of the aponeurosis (Figure 5), presumably due to the changes in the shape of the muscle belly and force transmission that are caused by the different aponeurosis properties.

In our simulations the aponeurosis was included in an unrealistically thick (3 mm) state for computational simplicity, however the material properties of the aponeurosis was scaled so that its overall stiffness matched that expected for the *in vivo* condition. However, the thickness in the aponeurosis in our simulations allowed us to observe gradients of stress in the thickness direction (y-direction) that change with the aponeurosis stiffness. The simulations showed a reduced muscle fiber strain near the ends of the muscle where the fibers are structurally close to the fixed-end boundary condition and pull against the stiff aponeurosis. It is possible that removing the fixed boundary constraints, for instance by including the external tendon, will reduce this effect in future studies. Our *in silica* results show that the stress in the aponeurosis increased for the stiffest conditions, with the highest stress being more concentrated toward the outer layers of the aponeurosis (Figure 5), and it is possible that this indicates increases in the risk of injury initiation at areas closer to the outer surface of the aponeurotic sheets.

The results from this study highlight that the mechanical output of a whole muscle should not simply be considered to be a scaled-up muscle fiber that matches the size of the whole muscle (Wakeling and Lee, 2011), or a simple sum of actions of all the individual muscle fibers (Hill, 1970), but instead depends on the complex interactions between the muscle fibers and connective tissues that is brought about by the 3D structure of the muscle. In particular we show how the effect of regionalizing the muscle activity to a particular volume of muscle fibers causes changes to both the magnitude and direction of the whole muscle force, even when the mean level of muscle activity remains unchanged.

In summary, our simulations indicate that muscles with stiffer aponeuroses would result in smaller aponeurosis stretches and muscle fiber shortening. This effect would place the muscle fibers at a longer length on the ascending limb of their force-length curves, allowing them to develop greater stress and force. Additionally, as the stretch in the aponeurosis is reduced, the muscle fibers did not increase in their pennation angle as much during contraction. The simulations of regionalized and non-uniform activation patterns caused local differences in the shape of the muscle belly, strains and orientations of the muscle fibers. These factors affect both the magnitude and direction of the resultant muscle force.

## Funding

We gratefully acknowledge funding from Natural Sciences and Engineering Research Council of Canada (Nilima Nigam and James M. Wakeling) and the Canada Research Chairs Program (Nilima Nigam).

### Conflict of interest statement

The authors declare that the research was conducted in the absence of any commercial or financial relationships that could be construed as a potential conflict of interest.
